# De novo transcriptome analysis of *Viola* ×*wittrockiana* exposed to high temperature stress

**DOI:** 10.1371/journal.pone.0222344

**Published:** 2019-09-24

**Authors:** Xiaohua Du, Xiaopei Zhu, Yaping Yang, Yanli Wang, Paul Arens, Huichao Liu

**Affiliations:** 1 School of Horticulture and Landscape Architecture, Henan Institute of Science and Technology, Xinxiang, Henan, China; 2 Henan Province Engineering Research Center of Horticultural Plant Resource Utilization and Germplasm Enhancement, Xinxiang, Henan, China; 3 Wageningen University & Research, Wageningen, The Netherlands; Key Laboratory of Horticultural Plant Biology (MOE), CHINA

## Abstract

Around the world, pansies are one of the most popular garden flowers, but they are generally sensitive to high temperatures, and this limits the practicality of planting them during the warmest days of the year. However, a few pansy germplasms with improved heat tolerance have been discovered or bred, but the mechanisms of their heat resistance are not understood. In this study, we investigated the transcript profiles of a heat-tolerant pansy inbred line, DFM16, in response to high temperatures using RNAseq. Approximately 55.48 Gb of nucleotide data were obtained and assembled into 167,576 unigenes with an average length of 959 bp, of which, 5,708 genes were found to be differentially expressed after heat treatments. Real-time qPCR was performed to validate the expression profiles of the selected genes. Nine metabolic pathways were found to be significantly enriched, in the analysis of the differentially expressed genes. Several potentially interesting genes that encoded putative transcription regulators or key components involving heat shock protein (HSP), heat shock transcription factors (HSF), and antioxidants biosynthesis, were identified. These genes were highlighted to indicate their significance in response to heat stress and will be used as candidate genes to improve pansy heat-tolerance in the future.

## Introduction

Pansies (*Viola* ×*wittrockiana*) are among the most popular garden and landscape flowers, owing to their rich flower color and long flowering period. The ideal temperature for pansy growth and flowering ranges from about 14°C to 21°C [1, 2], and the main disadvantage is that they cannot endure beyond 30°C as the resulting heat stress affects their overall growth and bloom development.

Heat stress affect the development and growth of many plants in a variety of ways, such as reducing the percentage germination, retardation in shoot growth, leaf chlorosis, reducing pollen fertility, decreasing yields, and even death [3, 4]. These phenotypes have direct relationship with imbalances in the physiological processes of a plant. For example, heat stress disturbs the stability of cell membranes and causes reactive oxygen species (ROS) bursts, resulting in germination barriers, decreases in pollen fertility and damages to the plant [5, 6]. The inhibition of photosynthetic activity under heat stress conditions causes leaf etiolation [7].

To cope with high temperature stress, plants have evolved various tolerance mechanisms that may vary significantly from species to species. According to their heat tolerance, plants can be classified into one of three groups: heat-sensitive species, relatively heat-resistant species and heat-tolerant species [6]. Some major tolerance mechanisms include osmoprotectants, antioxidants, factors involved in signaling cascades, molecular chaperones and regulatory proteins [8–10]. Proline, glycine betaine, and trehalose, are well-known osmoprotectants that can accumulate in response to heat stress to stabilize and protect proteins and membranes and maintain water relations [11].

ROS are a leading response to oxidative stress and a consequence of heat stress. Tolerant plants possess powerful antioxidant capacities by the synthesis of antioxidant enzymes and antioxidant metabolites, such as ascorbate (AsA) and glutathione (GSH) that scavenge ROS and generate detoxification systems [12]. The major antioxidant enzymes include ascorbate peroxidase (APX), catalase (CAT), glutathione S-transferase (GST), superoxide dismutase (SOD), peroxidase (POX), and glutathione reductase (GR), which play important roles in heat stress tolerance [13]. Under heat stress, the activities of SOD, POX, CAT, APX, and GR in heat tolerant wheat cultivars increased significantly, while the activities of POX, CAT, and GR in susceptible cultivars decreased significantly [14].

Heat shock proteins (HSPs) function as molecular chaperones. They are strongly induced under heat stress and play critical role in protecting intracellular proteins from denaturation, by preserving their stability and function, through protein folding [15, 16]. Currently, five major HSPs families have been identified, including HSP100, HSP90, HSP70, HSP60, and small HSPs (sHSPs). The expression of these HSPs is regulated mainly by heat shock transcription factors (HSFs). HSFs serve as the terminal components of the heat stress response in the signal transduction pathway [17].

At the molecular level, heat stress causes alterations in the expression of stress-related proteins in sensing, signaling, and defending plant cells, as well as in the transcription of genes responsible for the expression of calcium sensors, receptor‑like protein kinases (RLKs), transporter proteins like aquaporins, signal transducers, transcription factors, and chaperones [18]. Plant responses to high temperature stress is more complex, because the response are dynamic, depending upon the extremity and duration of the situation, and the plant type [6]. This has meant that the mechanisms of plant responses to heat stress have remained elusive. Investigations of these underlying molecular processes may shed light on the mechanisms of heat resistance in plants and help to develop heat-tolerant cultivars [19].

Pansies would be planted outdoors during the warmest days of the year, if they could tolerate the conditions. Therefore, breeding heat-tolerant cultivars has been a focus of research in recent years. A pansy inbred line, DFM16, appears to express stable levels of heat tolerance. It would be helpful, for the genetic improvement of heat tolerance in pansies, to understand the key genes, and the underlying molecular mechanisms in the DFM16 germplasm material. However, the underlying molecular mechanisms of heat tolerance in pansies remain poorly understood, due to there being very few studies and only limited genetic and sequence information available. With next generation sequencing developments, RNA-Seq technology has provided a comprehensive and efficient way to obtain functional genes and analyze transcriptomes, especially for non-model organisms without genomic sequences. In this study, DFM16, a heat-tolerant pansy germplasm, was de novo sequenced at the transcriptome level under heat stress conditions, using the Illumina platform, to investigate the resulting molecular mechanisms. The present study provides genomic resource data for gene discovery, and the identification of potential genes related to high temperature regulations that will be helpful in developing more heat-tolerant pansies in the future, through genetic engineering.

## Materials and methods

### Plant materials

**Fig 1:**
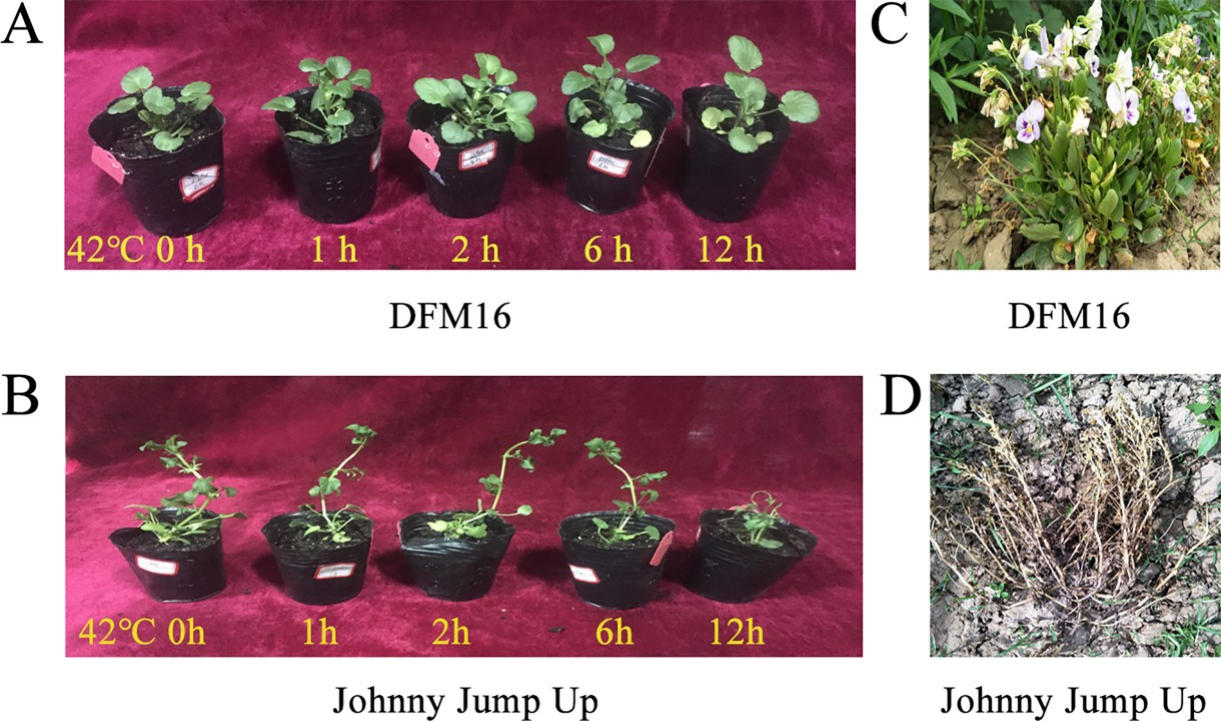
DFM16 showed heat tolerance phenotype compare to ‘Johnny Jump Up’. (A) The appearance of 60-day-old seedlings of DFM16 after exposure to 42°C for 0, 1, 2, 6 and 12 h. (B) The appearance of 60-day-old seedlings of ‘Johnny Jump Up’ seedlings exposed to 42°C for 0, 1, 2, 6 and 12 h. (C) The performance of adult plants of DFM16 on June 12, 2019, growing at the experimental field of Henan Institute of Science and Technology, Xinxiang (35° N, 113° E), China. (D) The performance of adult plants of ‘Johnny Jump Up’ on June 12, 2019, growing at the same site as DFM16.

A pansy inbred line, DFM16, derived from a commercial cultivar of *V*. ×*wittrockiana* ‘Frühblühende Mischung’ after four generations of artificial selfing and selection by the Pansy Breeding Group of Henan Institute of Science and Technology, exhibiting a heat tolerant phenotype both in the field and under control condition (Fig 1), was used for RNA-Seq. ‘Johnny Jump Up’, a widely cultivated pansy, was used as a control for the determination of heat tolerance of DFM16 through physiological indices measurements. The sterile seeds were potted into a 1:1 (*v*/*v*) mixture of peat and vermiculite and grew in an artificial climate incubator (Taihong Zhujiang LRH-250-GB, Guangzhou, China) with 50% of relative humidity, 7000 lux light intensity, 14 h photoperiod, and 20°C for 60 d.

### Measurement of physiological indices for heat tolerance

The 60-day-old plants of two lines were treated with 42/37°C (day/night) and 25/20°C (day/night) respectively for 4 days. Each treatment included 10 plants per line.

Electrolyte leakage was measured according to the method described by Zou [20], using a conductivity meter DDS-11A (Shanghai Instrument and Electric Science Instrument Co., Ltd., Shanghai, China). Photochemical efficiency (Fv/Fm) was measured by a Chl-Fluorescence Analyzer Yaxin-1161 (Beijing Yaxin Liyi Science Technology Co., Ltd., Beijing, China) according to the manufacturer's instructions. Proline concentration was estimated by the ninhydrin method following Song *et al*. [21]. The activity of POD and CAT were determined from the absorbance change at 470 nm caused by the oxidation of guaiacol according to the method described by Zou [20]. Each assay was replicated three times.

### RNA quantification and qualification

Healthy and uniform 60-day-old seedlings of DFM16 were selected and randomly assigned to three separate groups that were incubated at 25°C (control group), 42°C for 1 h, 42°C for 2 h, in an artificial climate incubator (Taihong Zhujiang LRH-250-GB, Guangzhou, China) with 50% of relative humidity and 7000 lux light intensity. Each group had two replicates, and each replicate had five seedlings. For each seedling, the top three to four leaves were collected. The collected leaves from the five seedlings in each replicate were mixed and frozen immediately in liquid nitrogen, and then stored at −80°C. The samples were divided into three groups with two biological replicates: control_1 and control_2, high temperature stress for 1 h (T1_1 and T1_2), and high temperature stress for 2 h (T2_1 and T2_2).

Total RNA was isolated from each sample using an RNAprep Pure Plant kit (TIANGEN Biotech, Beijing, China) according to the manufacturer’s instructions with some modifications. The RNA quality and purity were verified with a Nanodrop 2000 and electrophoresis on 1.0% agarose gels. RNA integrity (RIN value) was assessed using an Agilent 2100 Bioanalyzer (Agilent Technologies, CA, USA). RNAs with a RIN of more than 8.0, were used for further experiments.

### Library preparation for transcriptome sequencing

A total amount of 1.5 μg of RNA per sample was used as input material for the RNA sample preparations. cDNA libraries for sequencing were generated using NEBNext^®^ Ultra^™^ RNA Library Prep Kit for Illumina^®^ (NEB, USA) following the manufacturer’s recommendations, and index codes were added to attribute sequences to each sample. In order to select cDNA fragments that were approximately 150 ~ 200 bp in length, the library fragments were purified with the AMPure XP system (Beckman Coulter, Beverly, USA). Then PCR was performed, and the PCR products were purified. The products were then sequenced using an Illumina HiSeq2500 at Novogene Tech Co., Ltd.

### Sequence data analysis and annotation

Raw data (raw reads) of the fastq format were first processed through in-house Perl scripts. In this step, clean data (clean reads) were obtained by removing reads containing adaptors, reads containing poly-N, and low-quality reads. At the same time, Q20, Q30, GC-content and sequence duplication level of the clean data were calculated. The following analysis was based on clean data with high quality. Gene function was annotated based on the following databases: Nr (NCBI non-redundant protein sequences), Nt (NCBI non-redundant nucleotide sequences), Pfam (Protein family), KOG/COG (Clusters of Orthologous Groups of proteins), Swiss-Prot (a manually annotated and reviewed protein sequence database), KO (KEGG Ortholog database), and GO (Gene Ontology).

### Differentially expressed gene analysis

Gene expression levels for each sample were estimated by RNASeq by Expectation Maximization [22]. Differential expression analysis of the two groups was performed using the DESeq R package, which provided statistical routines for determining differential expression in digital gene expression data using a model based on the negative binomial distribution. DEGs were selected using the criteria of an adjusted *p*-value ≤ 0.05 and an absolute value of log2 ratio (treatment/control) ≥ 1.

GO enrichment analysis of the DEGs was implemented by the GOseq R packages based on Wallenius non-central hyper-geometric distribution [23], which can adjust for gene length bias in DEGs. KEGG pathway enrichment analysis of DEGs was carried out using KOBAS software [24].

### Quantitative real-time PCR validation

Quantitative real-time PCR (qRT-PCR) was performed to validate differential gene expression in terms of RNA-seq. Total RNA was separately extracted from the leaves after the plant was subjected to 42°C heat treatments, for 1 or 2 h, and leaves from untreated controls were as described above. First-strand cDNA was synthesized with the PrimeScript^®^ RT reagent kit (Takara Biotechnology, Dalian, China), according to the manufacturer’s instructions. The primers used in this study were designed with Primer premier 5.0 and listed in S1 Table. The *actin* gene was selected as an internal reference gene. qRT-PCR was performed using a CFX Connect Real-Time PCR System (Bio-rad Laboratories Inc, Singapore) using SYBR Green PCR Master Mix (TIANGEN Biotech, Beijing, China). The PCR program was as follows: pre-incubation at 95°C (3 min), 40 cycles of amplification for at 95°C (30 s), 60°C and 72°C (15 s). For each sample, the reactions were performed in triplicate, and the relative mRNA levels were calculated using the 2^−ΔΔCt^ method [25].

### Statistical analysis

The data were analyzed using one-way analysis of variance followed by the Tukey’s test to identify means differing significantly from one another on Data Processing System v7.55 software (Zhejiang University, Hangzhou, China).

## Results

### Determination of heat tolerance in DFM16

**Fig 2:**
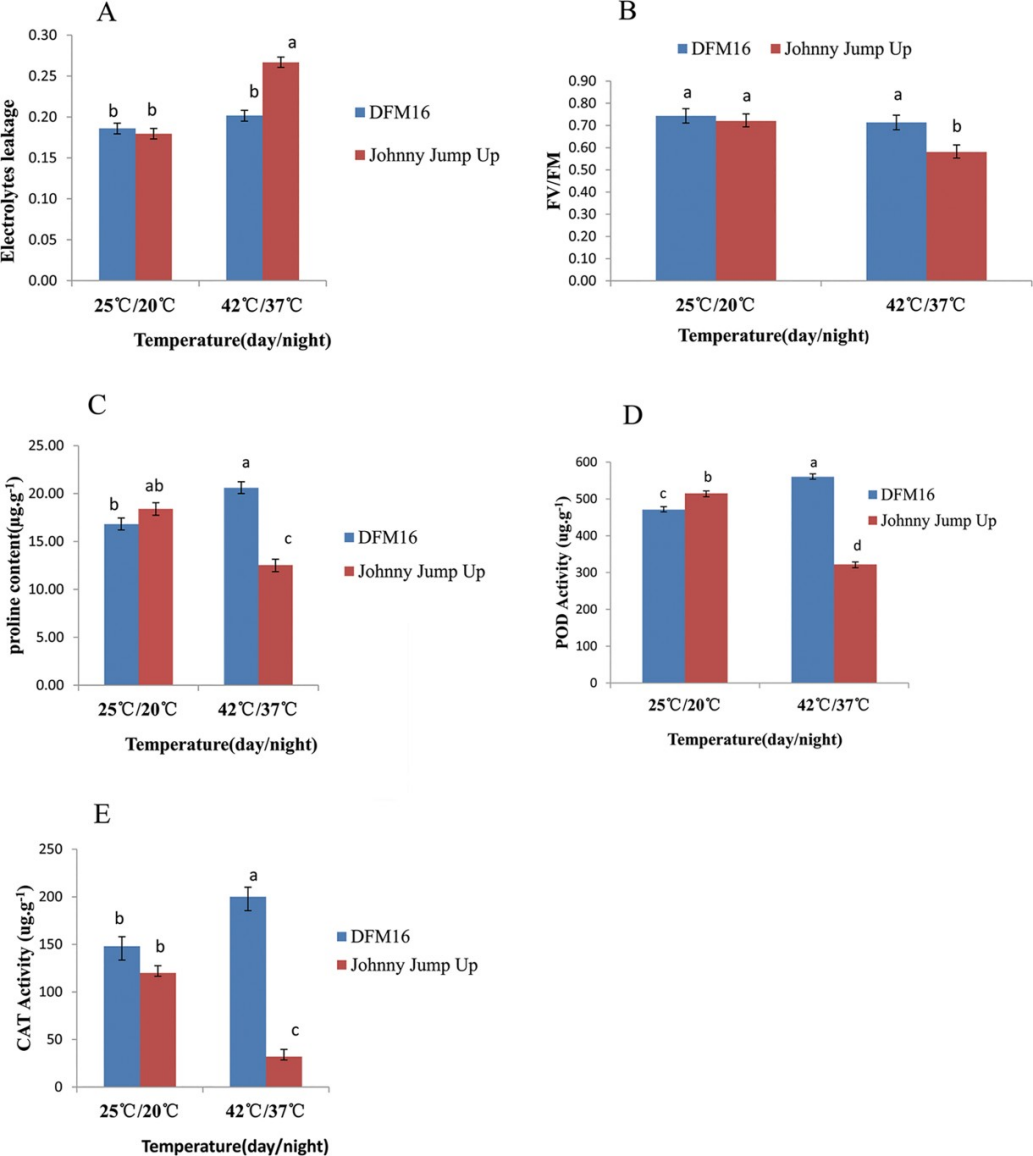
Comparison of the physiological indices of pansy DFM16 and ‘Johnny Jump Up’ under heat stress treatment. (A) Electrolyte leakage; (B) photochemical efficiency (Fv/Fm); (C) proline concentration; (D) POD activity; (E) CAT activity. Error bars are based on three replicates. Different letters show means differing from one another significantly at *p* ≤ 0.05.

The 60-day-old DFM16 seedlings expressed no significant changes in phenotype except for the bottom leaves yellowing after exposure to 42°C for 12 h (Fig 1A), while ‘Johnny Jump Up’ withered and died (Fig 1B). The electrolytes leakage of ‘Johnny Jump Up’ plants increased significantly after the plants grew at 42/37°C (day/night) for 4 d, compared with 25/20°C (day/night). While DFM16 plants showed no significant change in electrolytes leakage under heat stress (Fig 2A). These results indicated that the cell membrane of ‘Johnny Jump Up’ plant leaves was damaged by heat stress, while that of DFM16 was not. The Fv/Fm of plant leaves decreased significantly in ‘Johnny Jump Up’ but not in DFM16, after growing at 42/37°C (day/night) for 4 d (Fig 2B), which indicated that the photosynthetic system was damaged by heat stress in ‘Johnny Jump Up’ but not in DFM16. The proline concentration, POD activity and CAT activity increased in DFM16 but decreased significantly in ‘Johnny Jump Up’ under heat stress (Fig 2C–2E).

### Illumina sequencing and de novo assembly

To obtain the *V*. ×*wittrockiana* transcriptome responding to high temperature stress, six cDNA libraries were constructed from pansy leaves exposed to normal (control_1 and control_2) and high temperature (42°C) for 1 h (T1_1 and T1_2), and 2 h (T2_1 and T2_2), and they were sequenced using an Illumina HiSeq^TM^ 2500. The number of raw reads ranged from 51.48 to 80.64 for each sample. After filtering out the low-quality reads, there were 36.98 million clean reads, containing a total of 55.48 Gb nucleotides, obtained for all samples. The Q20 percentage (sequencing error rate < 1%) was greater than 95.09%, and the GC percentages for each sample were between 46.04 and 46.97% (Table 1). All clean reads from six individual libraries were pooled to perform the de novo assembly using the Trinity programs. Finally, a total of 167,576 unigenes were obtained, with an average length of 959 bp.

**Table 1:** Summary of pansy transcriptome sequencing in six samples.

Sample	Raw Reads	Clean Reads	Clean Bases (Gb)	Q20 (%)	GC Content (%)
control_1	64,803,708	63,549,658	9.53	95.44	46.04
control_2	56,826,144	55,450,342	8.32	96.7	46.14
T1_1	64,957,064	63,592,904	9.54	95.09	46.38
T1_2	51,488,846	50,143,334	7.52	96.49	46.46
T2_1	61,200,698	59,901,394	8.99	95.24	46.39
T2_2	80, 642,738	77,209,166	11.58	96.39	46.97

### Functional annotation and classification of the unigenes

**Fig 3:**
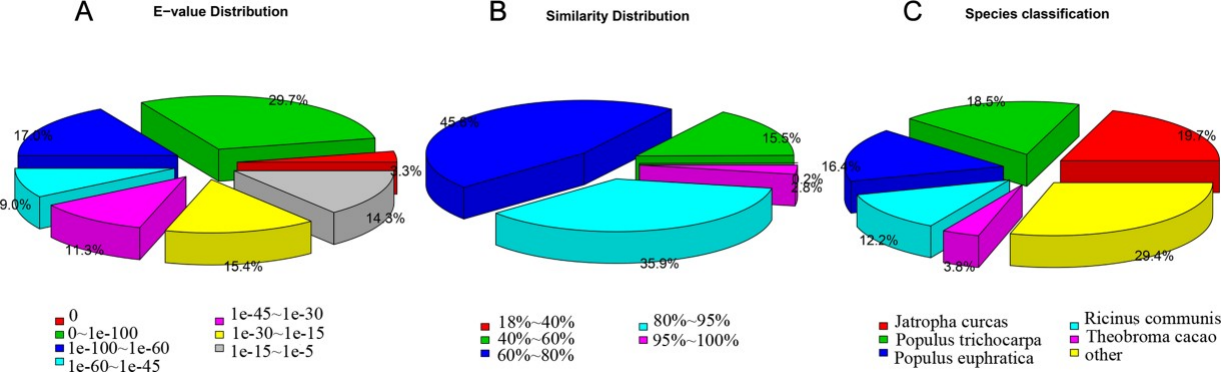
Characteristics of the homology search of *V*. *^×^wittrockiana* unigenes against the Nr database. (A) E-value distribution indicates the extent of sequence homology of unigenes annotated in the nr database. Different colors represent different range E-values. (B) Similarity distribution indicates the extent of sequence similarity of unigenes annotated in the nr database. Different colors represent different range values. (C) Species classification shows the distribution of the first BLAST hits for each sequence with a cut-off E-value of 1.0e^−5^. Different colors represent different species.

To annotate the assembled unigenes, BLAST searches were used in seven annotation databases: Nr, Nt, Swiss-Prot, Pfam, KOG, GO, and KEGG, with an E-value < 1e^−5^. The results indicated that among the 167,576 unigenes, 88,737 (52.95% of the total), 62,318 (37.18%), 67,466 (40.25%), 63,069 (37.63%), 22,807 (13.60%), 65,111 (38.85%), and 30,614 (18.27%), were significantly similar to known proteins or genes in the Nr, Nt, Swiss-Prot, Pfam, KOG, GO, and KEGG databases, respectively. Finally, a total of 101,509 (60.57%) unigenes were annotated, and 12 582 (7.50%) were found in all the databases (Table 2). Based on the annotation results of the Nr database, the E-value distribution analysis showed that 59% of matched sequences had strong homology, where the E-value < 1 e^−45^, and 41% sequences ranged between 1e^−5^ and 1e^−45^ (Fig 3A). The similarity distribution analysis revealed that 38.7% of the mapped sequences had higher than 80% similarity with the known sequence in the Nr database, and 45.6%of the sequences had a similarity ranging from 60 to 80% (Fig 3B). For species distribution, five species, *Jatropha curcas*, *Populus trichocarpa*, *Populus euphratica*, *Ricinus communis* and *Theobroma cacao* had the highest number of BLAST hits, with 19.7, 18.5, 16.4, 12.2, and 3.8%, respectively (Fig 3C).

**Table 2:** Summary for the annotation of unigenes of *V*. *^×^wittrockiana*.

Annotated databases	Number of Unigenes	Percentage (%)
NR	88,737	52.95
NT	62,318	37.18
SwissProt	67,466	40.25
Pfam	63,069	37.63
GO	65,111	38.85
KOG	22,807	13.60
KEGG	30,614	18.27
all Databases	12,582	7.50
at least one Database	101,509	60.57
Total Unigenes	167,576	100

**Fig 4:**
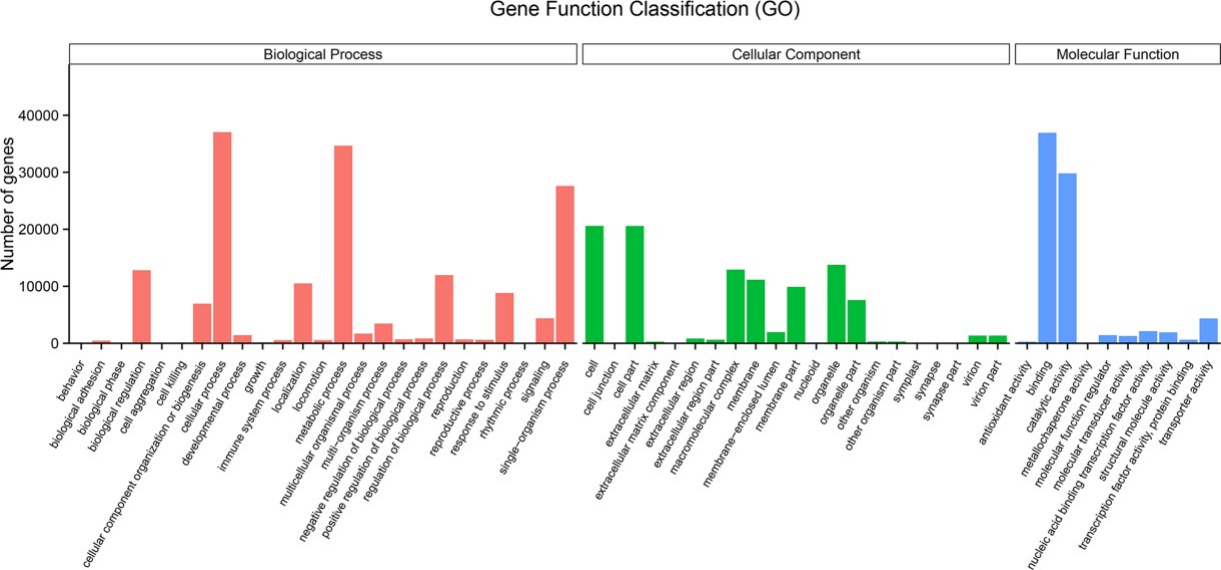
Gene function classification of *V. ^×^wittrockiana* unigenes. The GO categories included biological process (red), cellular component (green), and molecular function (blue). The x-axis indicates the main subcategory. The y-axis indicates the number of genes in the subcategory.

Based on the functional annotations and sequence homology, the predicted genes in the pansy were classified using the GO system. A total of 65,111 unigenes were assigned into three main categories: biological processes, cellular components, and molecular functions. The three main categories were further classified into 56 functional groups including 25 biological processes, 21 cellular components and 10 molecular functions (Fig 4). In the biological process category, “cellular process”, “metabolic process”, and “single-organism process”, were the most highly represented groups. For the cellular component, the major groups were “cell part” and “cell”. In the molecular function category, “binding” was dominant, followed by the group for “catalytic activity”. The GO analysis revealed that a great number of the identified unigenes were responsive to various biological processes, and cellular components in the *V*. *×wittrockiana* leaf tissues.

**Fig 5:**
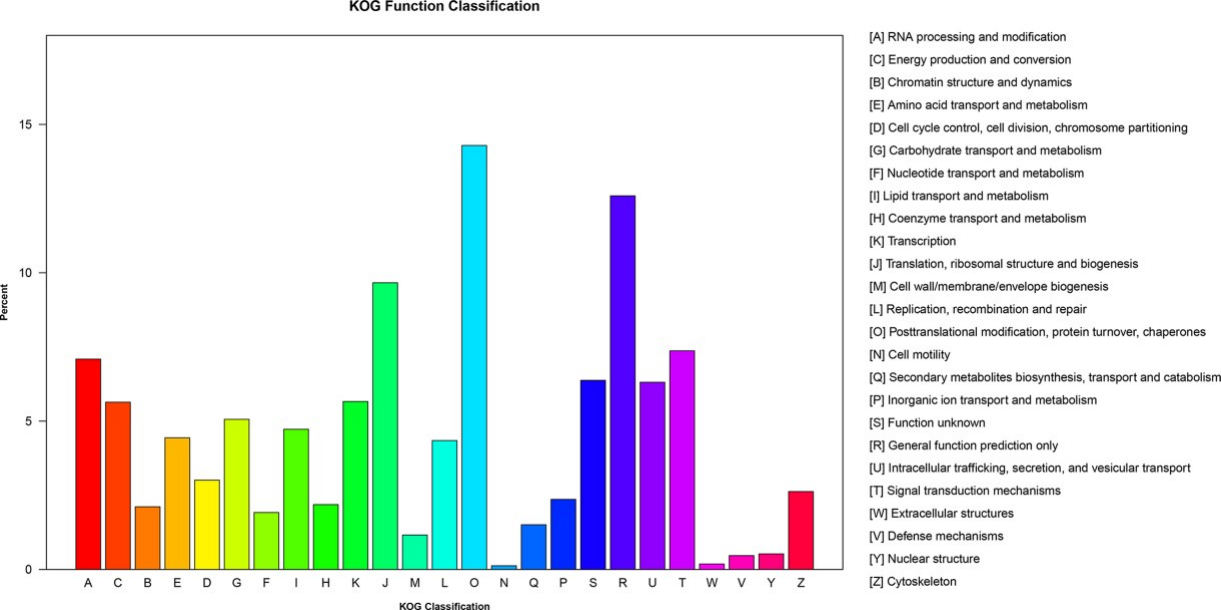
KOG classification of *V*. *^×^wittrockiana* unigenes. A total of 22, 807 unigenes show the significant homology (E-value < 1 e^−5^) to genes in one of the 25 categories (A-W, Y and Z) in the KOG database. The y-axis indicates the percent of gene numbers in the group.

The annotated sequences were further subjected to a search against the KOG database for functional prediction and phylogenetic classifications. As a result, 22,807 unigenes were grouped into 25 KOG classifications, among which the “post-translational modifications, protein turnover, and chaperones” represented the largest group (3,259 unigenes), followed by “General function prediction only” (2,872 unigenes), “Translation, ribosomal structure and biogenesis” (2,204 unigenes), “Signal transduction mechanisms” (1,681 unigenes), and “RNA processing and modifications” (1,617 unigenes) (Fig 5). The categories “Cell motility” and “Extracellular structures” with 29 and 42 unigenes were the smallest groups.

**Fig 6:**
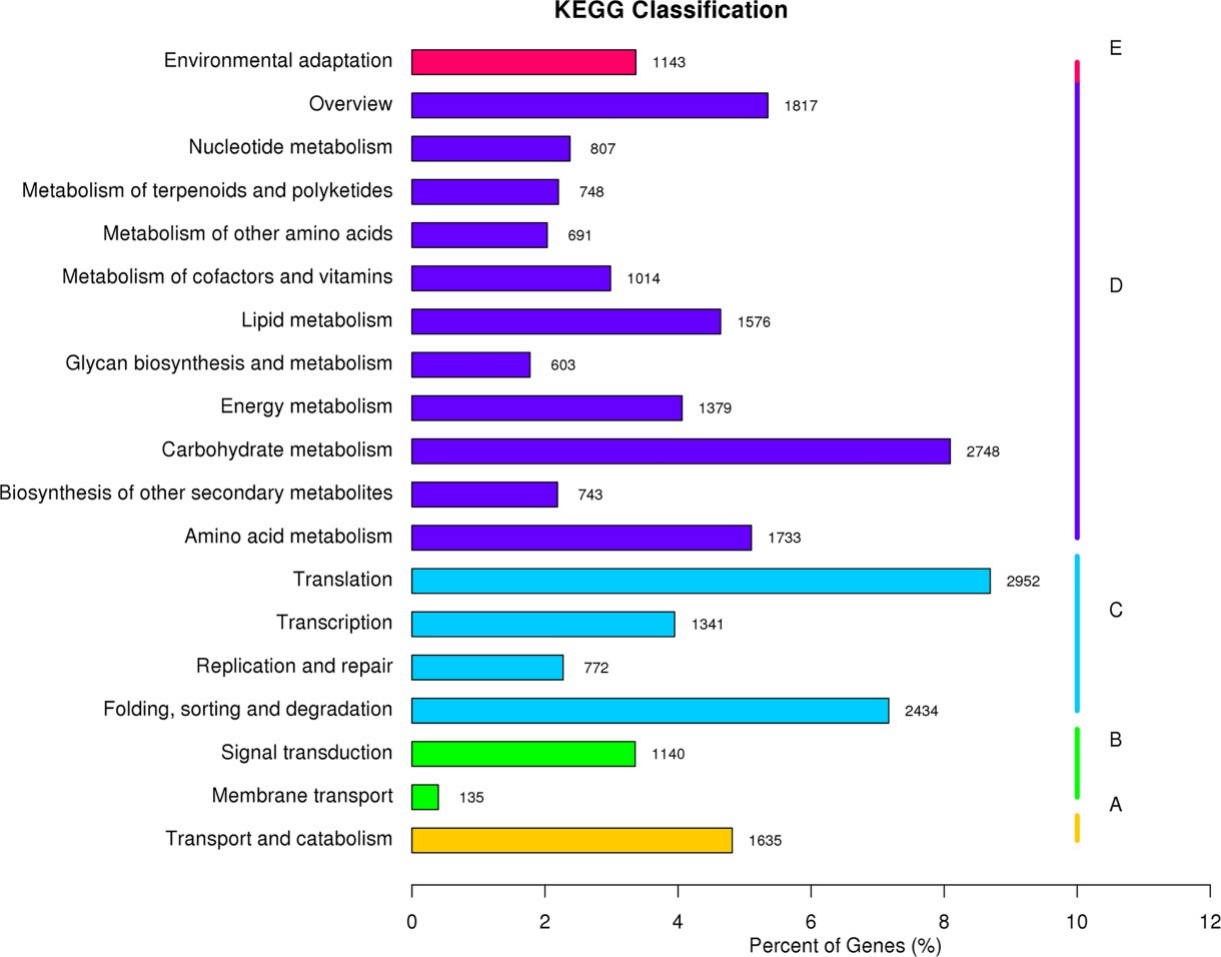
KEGG classification of *V*. *^×^wittrockiana* unigenes. The y-axis indicates the names of KEGG pathways. The x-axis indicates the number and percent of genes in the pathway. All the unigenes are summarized in five groups: A (Cellular Processes), B (Environmental Information Processing), C (Genetic Information Processing), D (Metabolism), E (Organismal Systems).

To obtain a better understanding of the biological functions of the unigenes, the annotated sequences were searched against the KEGG database. Among the 167,576 annotated unigenes, 30,614 had significant matches and were assigned to 19 KEGG pathways. The top five pathways were “Translation” (2,952 unigenes), “Carbon metabolism” (2,748 unigenes), “Folding, sorting and degradation” (2,434 unigenes), “Overview” (1,817 unigenes) and “Amino acid metabolism” (1,733 unigenes) (Fig 6). In summary, these annotation and classification analyses provide valuable information for gene discovery and functional genomic studies in the future.

### Identification of differential expressed genes (DEGs)

**Fig 7:**
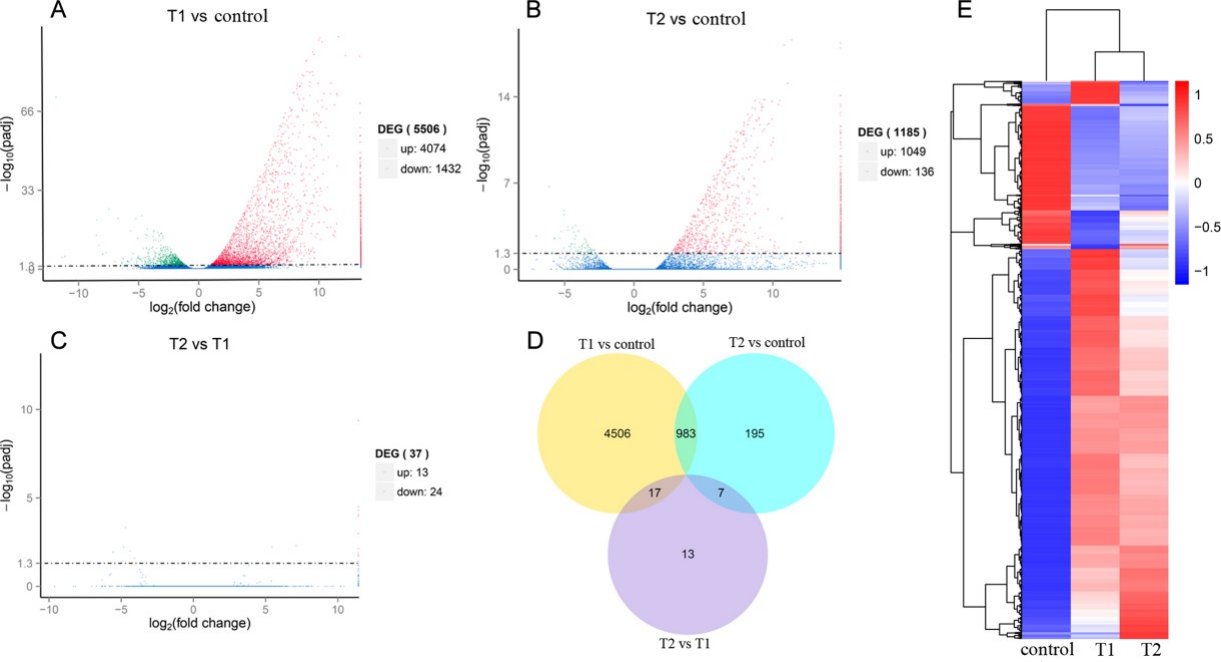
The DEG number and clustering analysis of DEG expression profile. (A-C) Volcano plots of the DEGs in the T1 vs control, T2 vs control and T2 vs T1 groups. Statistical significance (log_10_ (*p*-value)) (Y-axis) has been plotted against log_2_ (fold change) (X-axis). (D) Venn diagram of the DEGs, which shows the number of DEGs in the T1 vs control, T2 vs control and T2 vs T1 groups. (E) Heat map of the DEGs in the control, heat-treated sample at 1 h and 2 h. Each column represents one sample, and each row represents one DEG. Red indicates increased expression, and blue indicates decreased expression.

A total of 5,708 genes were identified that were differentially expressed under the different temperature treatments. The highest number of DEGs occurred after the heat treatment for 1 h, where a total of 4,074 genes were promptly up-regulated while 1,432 genes were down-regulated. When the heat treatment lasted for 2 h, 1,185 DEGs were observed, of which 1,049 genes were up-regulated, and 136 genes were down-regulated (Fig 7A and 7B). However, only 37 differential expression genes were identified in the T2 vs T1 pairwise comparisons (Fig 7C), of which 13 were up-regulated and 24 were down-regulated. Venn diagram illustrated that the groups T1 vs control and T2 vs control had 983 unigenes in common, but the group comparisons of T1 vs control and T2 vs T1, and the groups T2 vs control and T2 vs T1, had only 17 and 7 unigenes in common, respectively. This indicated that some genes may be maintained and only have differential expression changes in response to 2 h of heat exposure. However, these groups had no unigenes in common (Fig 7D). A hierarchical cluster analysis of the expression profiles of these DEGs was performed. Based on their expression patterns, they could be grouped into five classes. Genes in the first class were quickly up-regulated after 1 h, and then returned to a normal level at 2 h, whereas those in the second class were all down-regulated after 1 and 2 h. Genes in the third class were down-regulated after 1 h and up-regulated after 2 h. The fourth class they were up-regulated after 1 h and then slightly lowered but were still more than control at 2 h, and the fifth class they were upregulated at 1 h and further up-regulated at 2 h (Fig 7E).

### GO classification of DEGs

There were 3576 DEGs identified in response to the heat stress for 1 h. To generate an overview of these DEGs, the enriched GO terms of these DEGs were demonstrated (Table 3). The results showed that there were 39 significantly enriched GO terms (*p* < 0.01) for the heat responsive DEGs at 1 h. In the “cellular component” category, the most significant subcategories were “transcription factor complex” (GO: 0005667). In the “molecular function” category, “heme binding” (GO: 0020037) and “tetrapyrrole binding” (GO: 0046906) were significantly enriched subcategories. In the “biological process” category, the top three were “response to heat” (GO: 0009408), “ER overload response” (GO: 0006983) and “ER-nucleus signaling pathway” (GO: 0006984).

**Table 3:** Enriched GO terms of DEGs during heat treatment for different hours.

	GO ID	Description	Corrected *p*-value	
			T1 vs control	T2 vs control
Cellular component	GO:0005667	transcription factor complex	3.59E−06	
Molecular function	GO:0020037	heme binding	2.48E−11	
	GO:0046906	tetrapyrrole binding	2.85E−11	
	GO:0001671	ATPase activator activity	2.72E−09	0.0099191
	GO:0016491	oxidoreductase activity	2.98E−08	0.00021887
	GO:0001071	nucleic acid binding transcription factor activity	3.21E−07	
	GO:0003700	transcription factor activity, sequence-specific DNA binding	3.21E−07	
	GO:0051087	chaperone binding	3.21E−07	
	GO:0043565	sequence-specific DNA binding	1.25E−06	0.0010121
	GO:0016705	oxidoreductase activity, acting on paired donors, with incorporation or reduction of molecular oxygen	1.01E−05	
	GO:0005506	iron ion binding	2.03E−05	
	GO:0004601	peroxidase activity	8.43E−05	0.0099191
	GO:0016684	oxidoreductase activity, acting on peroxide as acceptor	0.00010098	
	GO:0015662	ATPase activity, coupled to transmembrane movement of ions, phosphorylative mechanism	0.0005409	
	GO:0051082	unfolded protein binding	0.00069045	
	GO:0008556	potassium-transporting ATPase activity	0.0010755	
	GO:0031072	heat shock protein binding	0.0012812	
	GO:0019829	cation-transporting ATPase activity	0.0034796	
	GO:0060590	ATPase regulator activity	0.0043675	
	GO:0003879	ATP phosphoribosyltransferase activity	0.0047359	
	GO:0016151	nickel cation binding	0.0061031	
	GO:0042625	ATPase activity, coupled to transmembrane movement of ions	0.0062819	
	GO:0004631	phosphomevalonate kinase activity		0.00047059
	GO:0016151	nickel cation binding		0.0027137
	GO:0004143	diacylglycerol kinase activity		0.0099191
Biological process	GO:0009408	response to heat	2.88E−09	
	GO:0006983	ER overload response	3.21E−07	
	GO:0006984	ER-nucleus signaling pathway	3.21E−07	
	GO:0071216	cellular response to biotic stimulus	3.21E−07	
	GO:0009266	response to temperature stimulus	2.59E−06	
	GO:0055114	oxidation-reduction process	6.63E−06	0.0020283
	GO:0000038	very long-chain fatty acid metabolic process	7.24E−06	
	GO:0034976	response to endoplasmic reticulum stress	6.47E−05	
	GO:0006804	obsolete peroxidase reaction	8.43E^−^05	0.0099191
	GO:0006979	response to oxidative stress	0.00020052	
	GO:0009414	response to water deprivation	0.00024163	
	GO:0032781	positive regulation of ATPase activity	0.00061586	
	GO:0019295	coenzyme M biosynthetic process	0.00064954	
	GO:0019296	coenzyme M metabolic process	0.00064954	
	GO:0043462	regulation of ATPase activity	0.00069045	
	GO:0009628	response to abiotic stimulus	0.00069045	
	GO:1901700	response to oxygen-containing compound	0.0017306	
	GO:0006695	cholesterol biosynthetic process		0.00038248
	GO:0008203	cholesterol metabolic process		0.00038248
	GO:1902652	secondary alcohol metabolic process		0.00038248
	GO:1902653	secondary alcohol biosynthetic process		0.00038248
	GO:0006120	mitochondrial electron transport, NADH to ubiquinone		0.0025678
	GO:0022904	respiratory electron transport chain		0.0099191
	GO:0007205	protein kinase C-activating G-protein coupled receptor signaling pathway		0.0099191
	GO:0042775	mitochondrial ATP synthesis coupled electron transport		0.0099191

Note: Only the very significantly enriched GO terms (*p* ≤ 0.01) in T1 vs control or/and T2 vs control were listed in this table.

After the heat treatment for 2 h, 758 DEGs were involved in 17 significantly enriched GO terms (*p* < 0.01). Compared with the enriched GO terms at 1 h, 6 GO terms were enriched at 1 h and 2 h, and 11 GO terms were enriched only in response to the heat stress after 2 h. In the cellular component categories, there were no significantly enriched subcategories. In the molecular function categories, the significant subcategories were “oxidoreductase activity” (GO: 0016491)), “phosphomevalonate kinase activity” (GO: 0004631) and “sequence-specific DNA binding” (GO: 0043565). In the “biological process” category, the top three were “cholesterol biosynthetic process” (GO: 0006695) “cholesterol metabolic process” (GO: 0008203), and “secondary alcohol metabolic process” (GO: 1902652).

### Pathway analysis of DEGs

The KEGG is a database resource for the systematic understanding of biological processes at molecular level. To further understand how the DEGs respond to high temperatures in the pansy, the DEGs were assigned to the terms in the KEGG database using BLASTx. The results showed that there were 1539 DEGs in the T1 sample and 338 DEGs in the T2 sample, mapped to KEGG pathways. Nine pathways, in total, were significantly enriched (*p* < 0.05) after heat treatments for 1 h. Among them, protein processing in the endoplasmic reticulum was the most significant (ko04141). When the high temperature treatment duration was extended from 1 h to 2 h, the protein processing in the endoplasmic reticulum (ko04141), glutathione metabolism (ko00480), oxidative phosphorylation (ko00190) and ascorbate and aldarate metabolism (ko00053), were still significantly enriched, while brassinosteroid biosynthesis (ko00905), glycerolipid metabolism (ko00561), tropane, piperidine and pyridine alkaloid biosynthesis (ko00960), histidine metabolism (ko00340), and nicotinate and nicotinamide metabolism (ko00760) were not significantly enriched (Table 4). These results indicated that with the initial heat treatment, the pansy respond to the external stimuli, and responsive genes were transcribed and repaired the membrane lipid. However, these gradually disappeared with prolonged duration of heat stress.

**Table 4:** List of the enriched KEGG pathways compared with control group.

Pathway ID	Term	DEGs with pathway annotation		All genes with pathway annotation
		T1 vs. control	T2 vs. control	(30614)
		(1539)	(338)	
ko04141	Protein processing in endoplasmic reticulum	185(12.0%)**	50(14.8%)**	942(3.1%)
ko00480	Glutathione metabolism	48(3.1%)**	15(4.4%)**	233(0.8%)
ko00190	Oxidative phosphorylation	52(3.4%)**	32(9.5%)**	478(1.6%)
ko00905	Brassinosteroid biosynthesis	13(0.8%)**	1(0.3%)	59(0.2%)
ko00561	Glycerolipid metabolism	33(2.1%))**	7(2.1%)	282(0.9%)
ko00053	Ascorbate and aldarate metabolism	22(1.4%)*	12(3.6%)**	169(0.6%)
ko00960	Tropane, piperidine and pyridine alkaloid biosynthesis	15(1.0%)*	4(1.2%)	103(0.3%)
ko00340	Histidine metabolism	13(0.8%)*	1(0.3%)	84(0.3%)
ko00760	Nicotinate and nicotinamide metabolism	16(1.0%)*	2(0.6%)	123(0.4%)

Note

* indicated the significant difference (*p* ≤ 0.05)

** indicated the very significant difference (*p* ≤ 0.01).

### Important genes shared by heat stress

**Fig 8:**
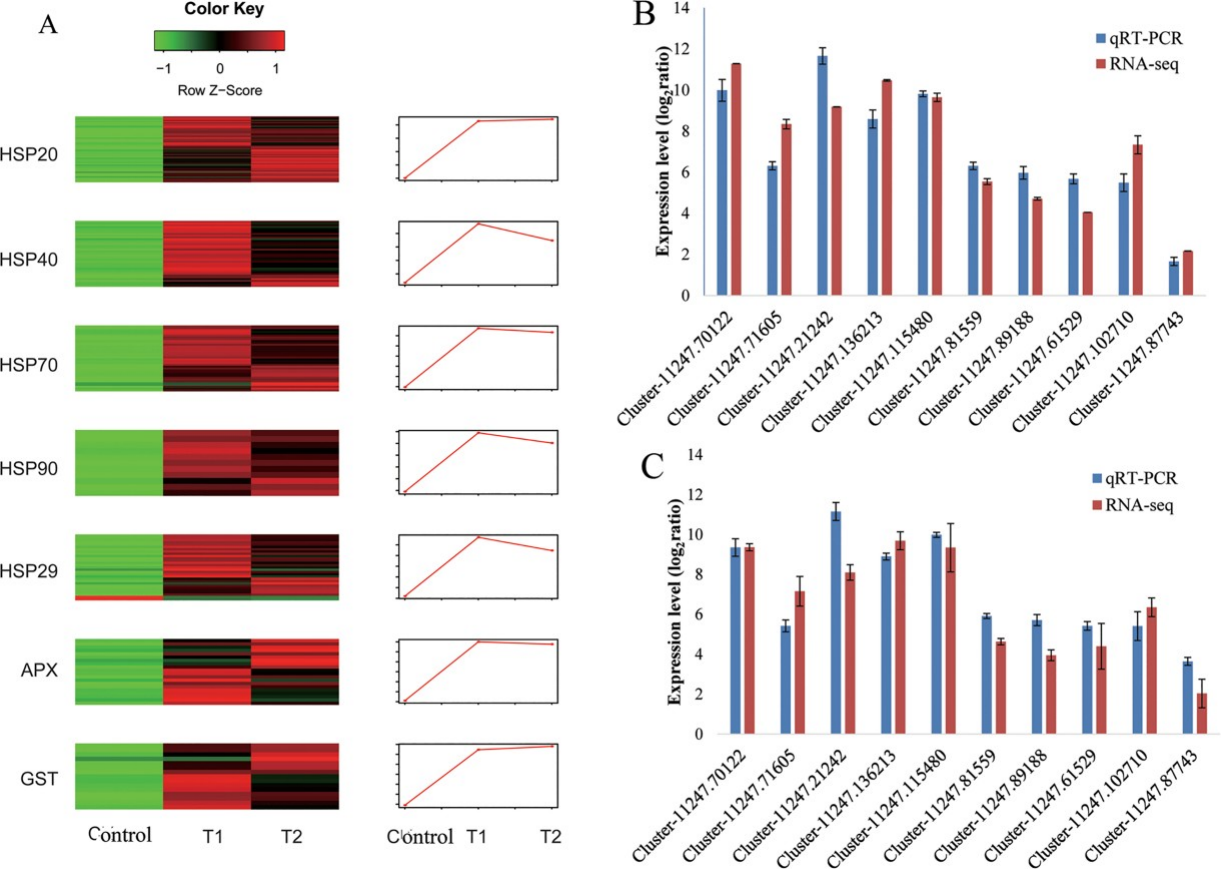
Heatmaps of DEGs from RNA-seq analysis and validation of ten DEGs by qRT-PCR analysis. (A) Heatmap of DEGs during the heat treatment. Each column represents one sample, and each row represents one DEG. Red indicates increased expression, and green indicates decreased expression. The line charts show gene expression profile cluster analysis of all genes in each group. (B, C) The comparison of the expression level of ten DEGs measured with qRT-PCR and RNA-seq. Bars indicate mean log_2_ (fold change) and whiskers indicate standard deviation. B: T1 vs control, C: T2 vs control.

HSFs were necessary for the induction of the heat stress response. HSPs and ROS-scavenging enzymes are major functional proteins induced by heat stress. In this study, 29 DEGs encoding HSFs, 20 DEGs encoding APXs and 141 *HSP* DEGs containing 56 *sHSP*, 38 *HSP40*, 36 *HSP70*, and 11 *HSP90* genes were found after the heat treatment in *V*. *×wittrockiana* (Fig 8A).

Ten unigenes involving two *sHSP* genes (Cluster-11247.115480, Cluster-11247.81559), two *HSP70* genes (Cluster-11247.89188, Cluster-11247.61529), one *HSP40* gene (Cluster-11247.102710), one *HSP90* gene (Cluster-11247.87743), as well as two *HSF* genes (Cluster-11247.21242, Cluster-11247.136213) and two *APX* genes (Cluster-11247.70122, Cluster-11247.71605), were selected for qRT-PCR analysis to validate the DEG expression profiling obtained from the RNA-seq. The RT-PCR analysis results were identical to those from RNA-Seq for the selected genes (Fig 8B and 8C).

Based on their expression pattern, they could be grouped into four classes. Genes in the first class (1 *HSF*, 9 *APX*, 8 *sHSP*, and 1 *HSP40* genes) did not express under normal condition and were activated by heat stress. Genes in the second class (14 *HSF*, 7 *APX*, 36 *sHSP*, 13 *HSP40*, 22 *HSP70*, and 7 *HSP90* genes) were up-regulated after 1 h and further increased after 2 h of heat treatment. Genes in the third class (12 *HSF*, 4 *APX*, 12 *sHSP*, 24 *HSP40*, 14 *HSP70*, and 4 *HSP90* genes) were quickly up-regulated after 1 h and then slightly declined after 2 h, compared with those at 1 h. In addition, only two *HSF* DEGs were down-regulated by the heat treatment.

Cluster analysis of the gene expression profiles showed that the expression levels of the *sHSP* gene members were always up-regulated after 2 h of heat stress. However, other gene families appeared to increase first and then began to decrease when the duration of the heat treatment extended to 2 h.

## Discussion

Generally, most pansy cultivars are heat-sensitive, and grow poorly when environmental temperature exceeds 30°C. Therefore, breeding heat-tolerant cultivars has become an important goal in pansy breeding. DFM16 exhibited a high level of heat tolerance on hot days compared with other pansy inbred lines in our previous study [26], when planted in Xinxiang, China. DFM16 maintained growth until July, while most other lines withered and died by the beginning of June. The heat tolerance of DFM16 was verified by its phenotype and some physiological indices, including electrolyte leakage, Fv/Fm, proline concentration, and activity of POD and CAT under control conditions and heat stress. The results indicated that DFM16 was heat tolerant by increasing proline concentration and activity of POD and CAT, to avoid damage to the cell membrane and photosynthetic system caused by heat stress (Figs 1 and 2).

Understanding heat resistance mechanisms and the identification of the key genes in heat-tolerant pansy germplasm, is vital for breeding heat-resistant cultivars of pansies, using transgenic approaches. Prior to this study, no genomic data were available for *V*. *×wittrockiana*, and RNA-sequencing was deemed to be as a sensible and powerful tool for in-depth analysis at the molecular level. The pansy line DFM16 was characterized as having better heat tolerance than other pansy breeding lines, in both field and artificial control conditions. To investigate its heat tolerance mechanisms, DFM16 was used for de novo assembly using RNA-seq technology.

The differential expression analysis of the RNA-seq data revealed that more DEGs were identified after 1 h of heat treatment than after 2 h, indicating that the DFM16 responds quickly to high temperature stress. GO classification analysis showed that the GO terms involved in response to heat, abiotic stimulus, endoplasmic reticulum stress, peroxidase reaction, oxidative stress, and oxidation-reduction process changed very significantly after 1 h of high temperature stress (Table 3). This showed that the high temperatures accelerated biochemical reactions and biological processes in the pansy, and several genes were expressed immediately. When stress lasted for 2 h, many genes associated with secondary metabolism (GO: 0006695, GO: 0008203, GO: 1902652), respiratory electron transport chains (GO: 0006120, GO: 0022904, GO: 0042775), and stress receptors (GO: 0007205), were increased. These results indicated that, with prolonged stress, respiration and secondary metabolism of the pansy were increased. To cope with the stress, pansy DFM16 triggered the signal transduction pathway to express more heat-resistant genes.

KEGG analysis suggested that the DFM16 pansy responded fast and significantly to high temperatures in at least two pathways. First, many genes involved in protein processing in the endoplasmic reticulum (ko04141) were the most highly expressed, which could be because many unfolded and misfolded proteins were accumulated under adverse environmental conditions and the repair and processing of misfolded proteins by HSPs occurred [16]. This was confirmed in our results that showed that the expressions of the HSP family of genes were induced most significantly during heat stress. A total of 141 *HSP* DEGs, including 56 *sHSP*, 38 *HSP40*, 36 *HSP70*, and 11 *HSP90* genes, were screened. HSPs have been known to assist in protein refolding as molecular chaperones when the newly synthesized proteins were misfolded, and the existing proteins were denatured under stress conditions [27]. It is considered that the high and rapid up-regulation of *HSP* genes was a hallmark of the heat stress response [27]. The results showed that all *HSP* DEGs were highly upregulated during heat stress in the pansy. Some evidence from other studies also supported that HSPs are enhanced by high temperature tolerance in plants. For example, over-expression of *HSP* genes, including *sHSP* [28, 29], *HSP70* [30], and *HSP90* [31], can confer heat tolerance in transgenic plants. Therefore, the up-regulated HSP genes played an important role in the heat tolerance of the pansy.

HSF are the core regulators of stress responses [17]. To cope with various patterns of heat stress, plants have developed large HSF families. A total of 19 and 21 HSF members have been cloned in *Oryza sativa* L. and *Arabidopsis thaliana*, respectively [15, 32]. In this study, 29 DEGs encoding HSFs were identified, which indicated at least 29 HSF members in *V*. *×wittrockiana*. The expression patterns of these DEGs were mainly upregulated, but only two declined, when heat stress occurred. One possible explanation for the distinct expression patterns responding to the same signal is the versatile functions or redundancy of HSFs. Apart from heat stress, HSFs are involved in other abiotic and biotic stress responses and plant development [17, 33]. In addition, there may be feedback regulation among HSFs. It has been demonstrated that HsfBs regulate the heat stress response by repressing the activity of HsfA1s in a negative feedback loop [34].

Second, antioxidant defenses were induced, including the metabolism of antioxidant enzymes and antioxidant metabolites (ko00480: glutathione metabolism, ko00053: ascorbate and aldarate metabolism). This could have resulted from the response of DFM16 to cope with oxidative stress such as ROS, which were caused by heat stress. It was documented that high temperature stress results in plants producing excess ROS, such as H_2_O_2_, O_2_^–^and ^1^O_2_ in plant cells, leading to oxidative stress [35]. Severe oxidative stress can cause damage to the photosynthetic membrane systems and cell membranes, resulting in decreased photosynthetic efficiency, cell electrolyte exosmosis, and leaf yellowing. These phenomena were observed on pansies [2], but the degree of damage for the DFM16 pansies was less serious [Figs 1 and 2]. This could partly attribute antioxidant enzymes in DFM16 as antioxidant enzymes that can eliminate ROS to enhance the heat stress tolerance in plants [11–13]. Present study showed that the activity of POD increased in DFM16 under heat stress. These results are similar to the findings for wheat, where the antioxidant enzymes were found to increase significantly in heat tolerant cultivars under heat stress but decrease in the heat sensitive cultivar [14].

APX, as a major enzyme in ROS-scavenging mechanisms, is involved in the ascorbic acid (ASA) cycle reaction that detoxifies H_2_O_2_ [36]. Over-expression of *apx* genes in tomato and tobacco resulted in enhanced highemperature tolerance [37, 38]. The *apx*1 and *cat*2 mutants showed heat stress-sensitive phenotypes [39]. In our study, the expression levels of 20 members in the *APX* gene family increased with heat treatments, which mean that the *APX* genes were stimulated by heat stress in the pansy. These results indicated that the higher expression of these genes probably increases the heat tolerance of the pansy to cope with environmental stress.

## Conclusions

In this study, we analyzed the transcriptome of a heat-tolerant pansy variety in response to heat stress using RNA-seq. We identified 5,708 genes that were differentially expressed after the heat treatment. Nine metabolic pathways were significantly enriched, and some genes encoding HSF, HSP, and ascorbate peroxidase were highlighted, revealing their important role in heat stress response. The candidate genes will provide genetic resources for improving the heat-tolerance characteristics in pansy. These results could also help improve our general understanding of the molecular mechanisms involved in heat stress responses in plants.

## Supporting information

S1 Table The primer sequences of 10 unigenes for qRT-PCR. (DOCX)
