# Supplementary material for: De novo transcriptome analysis of Viola ×wittrockiana exposed to high temperature stress
Source: PLoS One. 2019 Sep 24;14(9):e0222344. doi: 10.1371/journal.pone.0222344 (PMC6759194; doi:10.1371/journal.pone.0222344)
Supplement: S1 Table — (DOCX) [file pone.0222344.s001.docx]

**S1 Table. The primer sequences of 10 unigenes for qRT-PCR**

| **Gene ID** | **Primer name** | **Sequence(5'to 3')** | **Sequence length (bp)** |
| --- | --- | --- | --- |
| Cluster-11247.70122 | 70122-Forward | CTCCACTCATGCTCCGTCTC | 100 |
|  | 70122-Reverse | CACCATCACCACCATCCAA |  |
| Cluster-11247.71605 | 71605-Forward | TACCCGAAAGTGAGCGAGGAG | 106 |
|  | 71605-Reverse | CGAGACGGAGCATGAGTGGA |  |
| Cluster-11247.21242 | 21242-Forward | GTTCCTCATCGTCGCCGTAC | 166 |
|  | 21242-Reverse | GCACCTCCTCTGCTCCAAG |  |
| Cluster-11247.136213 | 136213-Forward | TGGAGTTGGCGTGAAAGAAGAGGC | 142 |
|  | 136213-Reverse | AGTTTTGGTCAGGAACGGAGGAGG |  |
| Cluster-11247.115480 | 115480-Forward | TCCTGAGCCCAAGAAACC | 114 |
|  | 115480-Reverse | CAAACATCACAGAACGAAAGAA |  |
| Cluster-11247.81559 | 81559-Forward | TAGGCCCGCCCATAATCT | 161 |
|  | 81559-Reverse | AGGTGGTGGTGCCGAAAG |  |
| Cluster-11247.89188 | 89188-Forward | TCTGAGAAGAAGCGACAA | 160 |
|  | 89188-Reverse | GTGAAAGCCACGATAGGAC |  |
| Cluster-11247.61529 | 61529-Forward | GAACTCAAGGATGCGATTA | 113 |
|  | 61529-Reverse | CCAGGTTGTTGGCTGTA |  |
| Cluster-11247.102710 | 102710-Forward | CCTGCTCCTCCATTACCATC | 166 |
|  | 102710-Reverse | CATCAAGGCTGTCCTATCCC |  |
| Cluster-11247.87743 | 87743-Forward | TCCCTGACAAGGCCAACAAC | 169 |
|  | 87743-Reverse | CCAACTCCAAACTGCCCAAT |  |
